# Neurotoxic Effects of 5-MeO-DIPT: A Psychoactive Tryptamine Derivative in Rats

**DOI:** 10.1007/s12640-016-9654-0

**Published:** 2016-07-26

**Authors:** Karolina Noworyta-Sokołowska, Katarzyna Kamińska, Grzegorz Kreiner, Zofia Rogóż, Krystyna Gołembiowska

**Affiliations:** 1 Department of Pharmacology, Institute of Pharmacology, Polish Academy of Sciences, 12 Smętna, 31-343 Kraków, Poland; 2 Department of Biochemistry, Polish Academy of Sciences, 12 Smętna, 31-343 Kraków, Poland

**Keywords:** 5-MeO-DIPT, DA, 5-HT, Glutamate in brain regions, DNA damage, Toxicity, Head twitch, Playing piano

## Abstract

5-Methoxy-N,N-diisopropyltryptamine (5-MeO-DIPT, ‘foxy’) is one of the most popular tryptamine hallucinogens in the illicit drug market. It produces serious adverse effects, but its pharmacological profile is not well recognized. In vitro data have shown that 5-MeO-DIPT acts as a potent serotonin transporter (SERT) inhibitor and displays high affinity at serotonin 5-HT1A, 5-HT2A, and 5-HT2C receptors. In this study, using microdialysis in freely moving rats, we examined the effect of 5-MeO-DIPT on dopamine (DA), serotonin (5-HT), and glutamate release in the rat striatum, nucleus accumbens, and frontal cortex. In search of a possible neurotoxic effect of 5-MeO-DIPT, we measured DA and 5-HT tissue content in the above rat brain regions and also determined the oxidative DNA damage with the comet assay. Moreover, we tested drug-elicited head-twitch response and a forepaw treading induced by 8-OH-DPAT. 5-MeO-DIPT at doses of 5, 10, and 20 mg/kg increased extracellular DA, 5-HT, and glutamate level but the differences in the potency were found between brain regions. 5-MeO-DIPT increased 5-HT and decreased 5-HIAA tissue content which seems to result from SERT inhibition. On the other hand, a decrease in DA, DOPAC, and HVA tissue contents suggests possible adaptive changes in DA turnover or damage of DA terminals by 5-MeO-DIPT. DNA single and double-strand breaks persisted up to 60 days after the treatment, indicating marked neurotoxicity of 5-MeO-DIPT. The induction of head-twitch response and potentiation of forepaw treading induced by 8-OH-DPAT indicate that hallucinogenic activity seems to be mediated through the stimulation of 5-HT2A and 5-HT1A receptors by 5-MeO-DIPT.

## Introduction

Hallucinogens are active substances that alter consciousness and affect the human psyche. Until now, we know relatively little about their mechanism of action in the brain. Despite their high degree of safety and lack of dependence liability (O’Brien 2001), hallucinogens have been labeled as the most dangerous drugs that exist, being placed into Schedule I of the Controlled Substances Act (CSA). Since September 29, 2004, 5-MeO-DIPT has been permanently controlled as a schedule I substance under the CSA (69 FR 58050) (DEA 2013), because it is used as a substitute for MDMA.

Classical hallucinogens may be divided into two broad categories: tryptamines, e.g., psilocybin, and phenethylamines, e.g., mescaline. Tryptamines comprise two groups of substances: simple tryptamines, such as DMT, 5-MeO-DMT, and ergolines, i.e., their relatively rigid analogues, such as LSD. Based on pharmacological, electrophysiological, and behavioral studies, it is hypothesized that classical hallucinogens produce their effects in animals and probably in humans primarily at cortical 5-HT2A receptor subtype (Aghajanian and Marek 1997, 1999; Glennon et al. 1984; Nelson et al. 1999; Nichols 1997; Scruggs et al. 2003; Smith et al. 1998, 1999; Sipes and Geyer. 1995). The activity of tryptamine hallucinogens was evidenced in drug discrimination studies conducted on rats. It was shown that 5-HT_2_ antagonists, like ketanserin and pirenperone blocked the discriminative stimulus effects of phenethylamine and tryptamine hallucinogens (Colpaert and Janssen 1983; Leysen et al. 1982). In addition, the head-twitch response (HTR) test is another animal model widely used to reliably distinguish hallucinogenic and nonhallucinogenic drugs the action of which is mediated by agonists of 5-HT2A receptors in mice and rats (González-Maeso et al. 2007). Schreiber et al. (1995) showed that head twitches induced by the phenylethylamine hallucinogen (±)DOI were abolished by low doses of the 5-HT2A-selective antagonist M100907, but not by the selective 5-HT2C antagonist, SB 200,646A. Mediation of behavioral effects induced by hallucinogens via 5-HT2A receptor is supported by electrophysiological and biochemical findings. Electrophysiological data demonstrated that stimulation of postsynaptic 5-HT2A receptors on pyramidal cells by hallucinogens led to glutamate-dependent increase in the activity of pyramidal neurons in layer V of the prefrontal cortex (Aghajanian and Marek 1997, 1999; Beique et al. 2007; Puig et al. 2003), while microdialysis studies showed enhancement of glutamate release by selective 5-HT2A agonist (±)DOI and LSD (Muschamp et al. 2004; Scruggs et al. 2003). Hallucinogens by acting at 5-HT2A receptors in the VTA may also activate brain DA pathways directly via somatodendritic receptors or presynaptic receptors in mesolimbic or mesocortical DA terminals. They may also affect DA pathways indirectly by modulating the GABA-ergic interneurons in the VTA (Celada et al. 2001; Vazquez-Borsetti et al. 2009). Besides 5-HT2A receptor activity, LSD, and tryptamines but not the phenethylamine-type hallucinogens, have high affinity for 5-HT1A receptors (deMontigny and Aghajanian 1977; Titeler et al. 1988). Administration of LSD, psilocybin, DMT, and 5-MeO-DMT caused a reduction in the firing rate of cells in the dorsal raphe nucleus (deMontigny and Aghajanian 1977). This observation led to the hypothesis that inhibition of 5-HT neuron activity via 5-HT1A autoreceptors might be the underlying mechanism for hallucinogenesis. However, 5-HT1A receptors, besides somatodendritic location, have a high postsynaptic density in limbic and cortical brain regions (Hamon et al. 1990; Pazos and Palacios 1985); and their stimulation leads to neuronal hyperpolarization (Hamon et al. 1990). In addition, it has been shown recently that 5-HT1A receptors are co-localized with 5-HT2A receptors on cortical pyramidal cells (Martin-Ruiz et al. 2001), where the two receptor types have opposing effects (Araneda and Andrade 1991). Willins and Meltzer (1997) reported that the 5-HT1A agonist 8-OH-DPAT inhibited (±)DOI-induced head twitches in rats. It was concluded that the activation of 5-HT1A receptors inhibited functional effects mediated by 5-HT2A receptors. Furthermore, most of the potent hallucinogenic compounds are also agonists of the 5-HT2C receptor (Chambers et al. 2001). Serotonin 5-HT2A and 5-HT2C receptors are both present on cortical GABA-ergic interneurons (Santana et al. 2004) and their activation has been observed to produce opposing behavioral effects (Fantegrossi et al. 2010). Moreover, the 5-HT2C receptors found in the VTA and nucleus accumbens exert tonic inhibitory action on mesolimbic and mesocortical dopaminergic neurons, and stimulation of those receptors suppresses DA release in the cortex and nucleus accumbens (De Deurwaerdere et al. 2004; Di Matteo et al. 1999). Thus, 5-HT2C receptors may play a modulatory role upon the control of dopaminergic-serotonergic-glutamatergic interactions in the mechanism of action of hallucinogens.

5-Methoxy-N,N-diisopropyltryptamine (5-MeO-DIPT), belonging to the tryptamine class of hallucinogens, in contrast to naturally occurring 5-MeO-DMT, DMT, or bufotenine, is a synthetic designer drug synthesized by Shulgin (1980). 5-MeO-DIPT, a popular illicit drug with a street name “foxy” or “foxy methoxy” is taken alone or mixed with other stimulants, e.g., with MDMA as ecstasy tablets (DEA 2013). It has been demonstrated that 5-MeO-DIPT is a competitive serotonin transporter (SERT) inhibitor and has a lower affinity for dopamine transporter (DAT) (Nagai et al. 2007; Sogawa et al. 2007). Fantegrossi et al. (2006) reported that hallucinogenic activity of 5-MeO-DIPT in mice was caused by the stimulation of postsynaptic 5-HT2A receptors, but 5-MeO-DIPT had also high affinity for 5-HT1A or 5-HT2C receptors as shown in vitro by Blough et al. (2014). 5-MeO-DIPT induced head-twitch responses in the mouse, and this effect was antagonized by the selective 5-HT2A receptor antagonist M100907 (Fantegrossi et al. 2006). Sogawa et al. (2007) demonstrated a marked cytotoxicity of 5-MeO-DIPT at high concentrations, as assessed by a cell viability assay in COS-7 cells. In another in vitro study, sustained exposure to 5-MeO-DIPT markedly decreased the intracellular 5-HT content in the mesencephalic slice culture (Nakagawa and Kaneko 2008). Clinical data indicated potent multi-organ effects of 5-MeO-DIPT as the users experienced euphoria, disinhibition, increased sociability, visual, and auditory hallucinations, but also effects like mioclonus, restlessness, insomnia and anxiety, nausea, vomiting, and diarrhea (Tittarelli et al. 2015). The possible toxicity of 5-MeO-DIPT is suggested by cognitive deficits observed in animals in some behavioral tests. It was found that 5-MeO-DIPT injected repeatedly to adolescent rats showed deleterious effects on learning and memory in adulthood (Compton et al. 2011; Skelton et al. 2009). Repeated doses of 5-MeO-DIPT altered ability of rats to perform certain cognitive tasks and caused hypoactivity and minor changes in 5-HT turnover in several brain regions (Williams et al. 2007).

Knowing the risk associated with 5-MeO-DIPT used recreationally and scarcity of in vivo data on the mechanism of its action in the brain, we examined the effects of 5-MeO-DIPT on neurotransmitter levels in several brain regions, and subsequent possible oxidative DNA damage. We also tried to show the 5-HT2A and 5-HT1A receptor effects of 5-MeO-DIPT in behavioral tests. Since the head-twitch response (HTR) is a behavior that is mediated by agonistic action at 5-HT2A receptors (González-Maeso et al. 2007), we tested the changes induced by several doses of 5-MeO-DIPT in the head-twitch response in comparison to the selective phenylethylamine 5-HT2A receptor agonist (±)DOI. Since binding data showed high affinity of 5-MeO-DIPT for 5-HT1A receptor, we also investigated 5-MeO-DIPT in vivo activity by using forepaw treading as the syndrome induced by 8-OH-DPAT and mediated via this receptor (Smith and Peroutka 1986).

## Materials and Methods

### Animals

The study was carried out on male Wistar-Han rats (Charles Rivers, Sulzfeld, Germany) weighing 280–300 g. The animals were housed in temperature- and humidity-controlled rooms under a 12-h light/12-h dark cycle, and had free access to standard laboratory food and tap water. The experiments were conducted in accordance with the European Union guidelines regarding the care and use of laboratory animals (Council Directive 86/609/EEC of November 24, 1986) and were approved by the II Local Bioethics Commission (Institute of Pharmacology, PAS, Kraków, Poland).

### Drugs and Reagents

5-Methoxy-N,N-diisopropyltryptamine (5-MeO-DIPT), 3,4- methylenedioxymethamphetamine (MDMA), were purchased from Toronto Research Chemicals Inc. (Canada). (1-(2,5-dimethoxy-4-iodophenyl)-aminopropane) hydrochloride (±)DOI), 8-hydroxy-2-(di-N-propylamino) tetralin (8-OH-DPAT) came from Sigma-Aldrich (Poland). The chemicals used for HPLC were obtained from Merck (Warsaw, Poland), while ketamine hydrochloride and xylazine hydrochloride came from Biowet (Puławy, Poland).

### Head-Twitch Test

Immediately after an injection of 5-MeO-DIPT (5–10 mg/kg sc) or (±)DOI (2.5 mg/kg ip), rats were placed individually in wire cages (21 cm width, 21 cm length, 25 cm high) and observation began. The number of head twitches was counted for a period of 30 min.

### Forepaw Treading Induced by 8-OH-DPAT

In the experimental group, 8-OH-DPAT (5 mg/kg ip) was given with 5-MeO-DIPT (5 or 10 mg/kg sc). The control group received 8-OH-DPAT (5 mg/kg ip). Immediately after the administration, each animal was separately placed in a wire cage (21 cm width, 21 cm length, 25 cm high). Observation began immediately after 8-OH-DPAT injection and lasted 30 min. The number of episodes of reciprocal forepaw treading was counted.

### Brain Microdialysis

Animals were anesthetized with ketamine (75 mg/kg) and xylazine (10 mg/kg), and vertical microdialysis probes were implanted into the striatum, nucleus accumbens, and frontal cortex using the following coordinates: AP +1.8, L +3.0, V −7.0; AP +1.6, L +1.1, V −8.0; AP +2.8, L +0.8, V −6.0 from the dura, respectively (Paxinos and Watson 1998). On the next day, probe inlets were connected to a syringe pump (BAS, IN, USA) which delivered artificial cerebrospinal fluid (aCSF) composed of [mM] NaCl 147, KCl 2.7, MgCl_2_ 1.0, CaCl_2_ 1.2; pH 7.4 at a flow rate of 2 µl/min. After 2 h of the washout period, three basal dialysate samples were collected every 20 min; then animals were injected subcutaneously with appropriate doses of 5-MeO-DIPT as indicated in figure captions and fraction collection continued for 240 min. At the end of the experiment, the rats were sacrificed and their brains were histologically examined to validate the probe placement.

### Analytical Procedure

DA and 5-HT were analyzed by high-performance liquid chromatography (HPLC) with coulochemical detection. Chromatography was performed using an Ultimate 3000 System (Dionex, USA), coulochemical detector Coulochem III (model 5300, ESA, USA) with 5020 guard cell, 5014B microdialysis cell, and Hypersil Gold C18 analytical column (3 μm, 3 × 100 mm). The mobile phase was composed of 0.1 M potassium phosphate buffer adjusted to pH 3.6, 0.5 mM Na_2_EDTA, 16 mg/L 1-octanesulfonic acid sodium salt, and 2 % methanol. The flow rate during analysis was set at 0.7 ml/min. The applied potential of a guard cell was 600 mV, while those of microdialysis cells were E1 = −50 mV, E2 = 300 mV with a sensitivity set at 50 nA/V. The chromatographic data were processed by Chromeleon v. 6.80 (Dionex, USA) software run on a PC computer.

Glutamate in extracellular fluid was measured electrochemically after derivatization with OPA/sulfite reagent to form isoindole-sulfonate derivative. Chromatography was performed using an LC-10 AD pump (Shimadzu Europa GmbH, Warsaw, Poland), an LC-4B amperometric detector with a cross-flow detector cell (BAS, IN, ISA), and a HR-80 column (80 × 4.6 mm, 3 μm; ESA, Inc. USA). The mobile phase consisted of 100 mM monosodium orthophosphate, 25 % methanol, pH 4.6. The flow rate was 0.9 ml/min, and the applied potential of a 3-mm glassy carbon electrode was +600 mV at a sensitivity of 5 nA/V. Glutamate-derivative peak was compared with the respective standard, and the data were processed using Chromax 2005 (Pol-Lab, Warszawa, Poland) software on a personal computer.

### The Tissue Content of DA, 5-HT and Their Metabolites

Animals were sacrificed by decapitation 4 h after subcutaneous drug administration. Brains were separated and several brain regions (striatum, nucleus accumbens septi, frontal cortex) were dissected in anatomical borders. The tissue levels of DA, 5-HT, DOPAC, HVA, and 5-HIAA were measured using a high-performance liquid chromatography (HPLC) with electrochemical detection. Briefly, tissue samples of brain structures were homogenized in an ice-cold 0.1 M HClO_4_ and were centrifuged at 10,000×*g* for 10 min at 4 °C. The supernatant (3–5 µL) was injected into a HPLC system. The chromatographic system consisted of an LC-4C amperometric detector with a cross-flow detector cell (BAS, IN, USA), an Ultimate 3000 pump (Thermo Scientific, USA) and a Hypersil Gold analytical column (3 μm, 100 × 3 mm, Thermo Scientific, USA). The mobile phase consisted of 0.1 M KH_2_PO_4_, 0.5 mM Na_2_EDTA, 80 mg/L sodium 1- octanesulfonate, and a 4 % methanol, adjusted to pH 3.7 with an 85 % H_3_PO_4_. The flow rate was 1 mL/min. The potential of a 3-mm glassy carbon electrode was set at 0.7 V with sensitivity of 5 nA/V. The temperature of the column was maintained at 30 °C. The Chromax 2007 program (Pol-Lab, Warszawa, Poland) was used for data collection and analysis.

### Comet Assay

#### Preparation of Nuclear Suspension

Animals were killed 3 or 60 days after termination of drug treatments. The whole cortex was separated in anatomical borders. Next, the brain tissue was minced with a surgical scalpel and homogenized in a manual homogenizer with homogenizing solution containing 0.25 % Triton. The homogenate was filtered and centrifuged at 850×*g* for 10 min. Thereafter the supernatant was discarded, while the pellet was resuspended in the same volume of homogenization medium without Triton and centrifuged for 10 min at 850×*g*. The sediment was washed once more in the same way and centrifuged at 600×*g* for 8 min. The pellet was resuspended in 0.8 ml of homogenization solution without Triton, mixed with 4.2 ml of purification medium and centrifuged at 19,000×*g* for 45 min. The nuclei were obtained as a transparent sediment at the bottom. The pellet was resuspended in 0.5 ml of 2.0 M sucrose and was layered over a sucrose gradient (2.6, 2.4 bottom to top). The gradient was allowed to stand for 3 h at 0 °C before use. Fractionation of the nuclei was achieved by centrifugation at 19,000×*g* for 45 min.

#### Alkaline Comet Assay

The nuclei were added to a tube with 200 µl of PBS (without Ca^++^ and Mg^++^) and mixed gently. The suspension was mixed with LMAagarose and transferred immediately onto Comet Slides. The slides were placed at 4 °C in the dark for 10 min. Then slides were immersed by prechilled Lysis Solution and left at 4 °C in the dark for 30 min. The buffer was drained, the slides were immersed in Alkaline Unwinding Solution and were left for 45 min in the dark. In the next step, electrophoresis was run at 21 V for 30 min. After electrophoresis, the slides were washed first with H_2_O, next with 70 % ethanol, and dried at 45 °C for 10 min. The slides were then covered with dye and allowed to dry completely at room temperature in the dark. On the next day, the slides were examined under a fluorescent microscope. DNA damage was presented as an olive tail moment. Olive tail moment is defined as the product of the tail length and the fraction of total DNA in the tail. Tail moment incorporates a measure of both the smallest detectable size of migrating DNA (reflected in the comet tail length) and the number of damaged pieces (represented by the intensity of DNA in the tail). The olive tail moment is calculated according to the formula: Olive Tail Moment = (Tail.mean−Head.mean) × Tail %DNA/100.

### Data Analysis

Repeated measures ANOVA followed by Tukey’s post hoc test were performed to analyze drug effect on DA, 5-HT, and glutamate release in the rat brain regions. Head-twitch response, forepaw treading, the effect of drug on tissue content of neurotransmitters, and DNA damage in comet assay were tested using one-way ANOVA followed by Tukey’s multiple comparison test. Differences were considered significant if *P* < 0.05. All statistical analyses were carried out using STATISTICA v.10 StatSoft, Inc. 1984–2011.

## Results

### The Effect of 5-MeO-DIPT on Head Twitches and Forepaw Treading in Rats

**Fig. 1:**
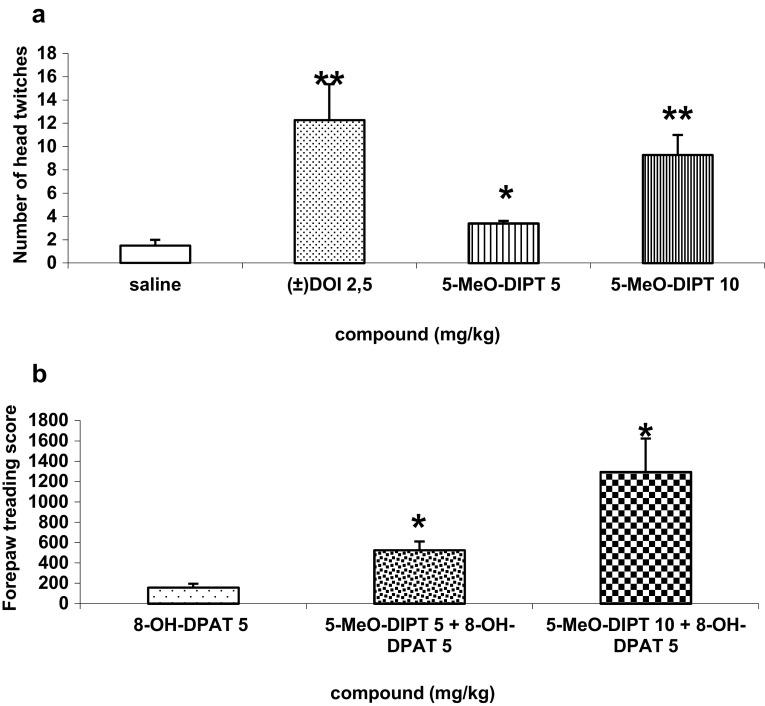
Head-twitch response induced by **a** 5-MeO-DIPT (5–10 mg/kg) and (±)DOI (2.5 mg/kg). *Bars* represent the number of head twitches (mean ± SEM of 7 animals per group) counted for 30 min starting immediately after injection. *P < 0.05, **P < 0.01 in comparison to saline. Forepaw treading induced by 8-OH-DPAT **b** 8-OH-DPAT (5 mg/kg), and 5-MeO-DIPT (5 or 10 mg/kg) were injected 3 min before the test. The results are presented as the mean ± SEM of 4–7 animals per group. * *P* < 0.01 in comparison to 8-OH-DPAT group

5-MeO-DIPT at doses of 5–10 mg/kg induced head twitches in rats, which were observed immediately after administration. The lower dose produced a weak effect but response to the higher dose was stronger and comparable to that of (±)DOI used as reference drug (Fig. 1a). Forepaw treading induced by 8-OH-DPAT (5 mg/kg) was significantly potentiated by both doses of 5-MeO-DIPT (Fig. 1b).

### The Effect of 5-MeO-DIPT on DA Release in the Rat Striatum, Nucleus Accumbens, and Frontal Cortex

The basal extracellular DA levels in the striatum, nucleus accumbens, and frontal cortex (in pg/10 μl) were 7.12 ± 0.66, 1.99 ± 0.22 and 0.29 ± 0.13, respectively and did not differ between experimental groups.

**Fig. 2:**
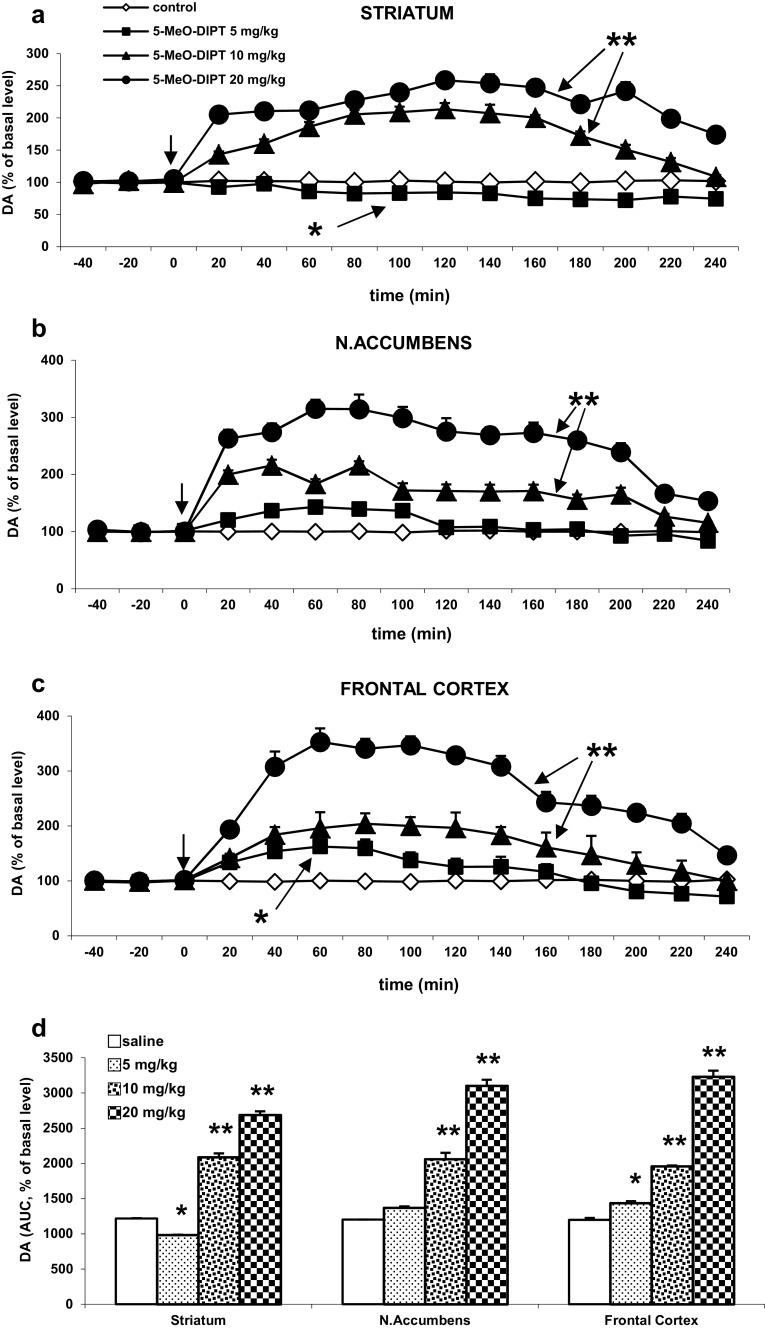
The effect of 5-MeO-DIPT (5, 10, and 20 mg/kg) on DA release in the rat striatum (**a**), nucleus accumbens (**b**), and frontal cortex (**c**). Time-course effect is shown. Data are presented as the mean ± SEM (*n* = 4–7). Drug administration is indicated with an *arrow*. The total effect of 5-MeO-DIPT on DA release (**d**) is expressed as an area under the curve (AUC) of a percent of the basal level. *P < 0.05, **P < 0.01 in comparison to control group (repeated measures ANOVA and Tukey’s post hoc test)

5-MeO-DIPT at doses of 10–20 mg/kg significantly increased DA release in the striatum to ca. 200–260 % (*P* < 0.01), but decreased it to ca. 75 % of the basal level at the lowest dose of 5 mg/kg (*P* < 0.05, Fig. 2a). Similarly, both higher doses of 5-MeO-DIPT but not the lowest one (5 mg/kg) significantly increased DA release in the nucleus accumbens (*P* < 0.01, Fig. 2b). In the frontal cortex, the effect of two higher doses of 5-MeO-DIPT was marked (*P* < 0.01) in enhancing extracellular DA level, while the lowest dose of 5 mg/kg weakly, but still significantly increased DA release (*P* < 0.05, Fig. 2c).

Repeated measures ANOVA showed a significant effect of treatment in the striatum F(3,18) = 322, *P* < 0, in the nucleus accumbens F(3,17) = 151, *P* < 0, and in the frontal cortex F(3,17) = 379, *P* < 0; significant effect of time F(11,198) = 17.5, *P* < 0 in the striatum, F(11,187) = 27.5, *P* < 0 in the nucleus accumbens, and F(11,187) = 75, *P* < 0 in the frontal cortex. Interaction between both factors was also significant in the striatum F(33,198) = 7.6, *P* < 0, in the nucleus accumbens F(33,187) = 6.6, *P* < 0, and in the frontal cortex F(33,187) = 16.4, *P* < 0.

The total time-course effect of DA release in all studied brain regions defined as an area under the curve (AUC) is presented in Fig. 2d. The total effect of higher 5-MeO-DIPT doses showed a significant increase in DA release, whereas the lower dose of 5 mg/kg decreased this release in the rat striatum and was without effect in the nucleus accumbens (Fig. 2d).

### The Effect of 5-MeO-DIPT on 5-HT Release in the Rat Striatum, Nucleus Accumbens, and Frontal Cortex

The basal extracellular 5-HT levels in the striatum, nucleus accumbens, and frontal cortex were (in pg/10 μl) 1.11 ± 0.24, 0.26 ± 0.06, 0.16 ± 0.01, respectively, and did not differ significantly between experimental groups.

**Fig. 3:**
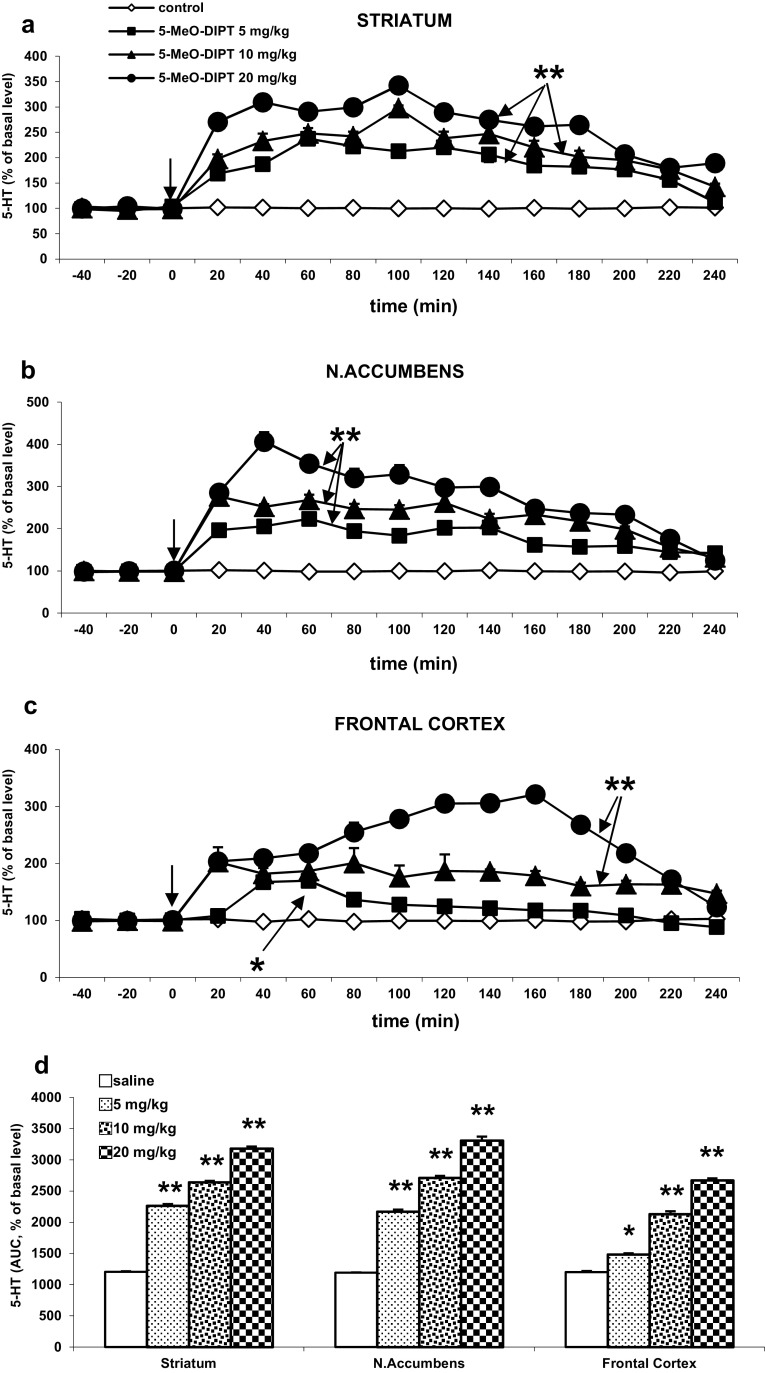
The effect of 5-MeO-DIPT (5, 10, and 20 mg/kg) on 5-HT release in the rat striatum (**a**), nucleus accumbens (**b**), and frontal cortex (**c**). Time-course effect is shown. Data are presented as the mean ± SEM (*n* = 4–7). Drug administration is indicated with an *arrow*. The total effect of 5-MeO-DIPT on 5-HT release (**d**) is expressed as an area under the curve (AUC) of a percent of the basal level. *P < 0.05, **P < 0.01 in comparison to control group (repeated measures ANOVA and Tukey’s post hoc test)

5-MeO-DIPT dose-dependently and significantly increased 5-HT release in the striatum, nucleus accumbens, and frontal cortex (*P* < 0.01, Fig. 3a, b, c). The 5-HT release enhancing effect at the lowest dose was the weakest but still significant in the frontal cortex (Fig. 3c).

Repeated measures ANOVA showed a significant effect of treatment in the striatum F(3, 21) = 290, *P* < 0, in the nucleus accumbens F(3,18) = 565, *P* < 0, and in the frontal cortex F(3,18) = 691, *P* < 0; significant effect of time F(11,231) = 33, *P* < 0 in the striatum, F(11,198) = 45, *P* < 0 in the nucleus accumbens, and F(11,198) = 15, *P* < 0 in the frontal cortex. Interaction between both factors was also significant in the striatum F(33,231) = 5.7, *P* < 0, in the nucleus accumbens F(33,198) = 10, *P* < 0, and in the frontal cortex F(33,198) = 9.8, *P* < 0.

The total time-course effect of 5-HT release in all studied brain regions defined as an area under the curve (AUC) is presented in Fig. 3d. The total effect of 5-MeO-DIPT at all doses showed a significant increase in 5-HT release.

### The Effect of 5-MeO-DIPT on Glutamate Release in the Rat Striatum, Nucleus Accumbens and Frontal Cortex

The basal extracellular glutamate levels in the striatum, nucleus accumbens, and frontal cortex were (in ng/10 μl) 2.12 ± 0.35, 2.15 ± 0.26, 2.70 ± 0.39, respectively, and did not differ significantly between experimental groups.

**Fig. 4:**
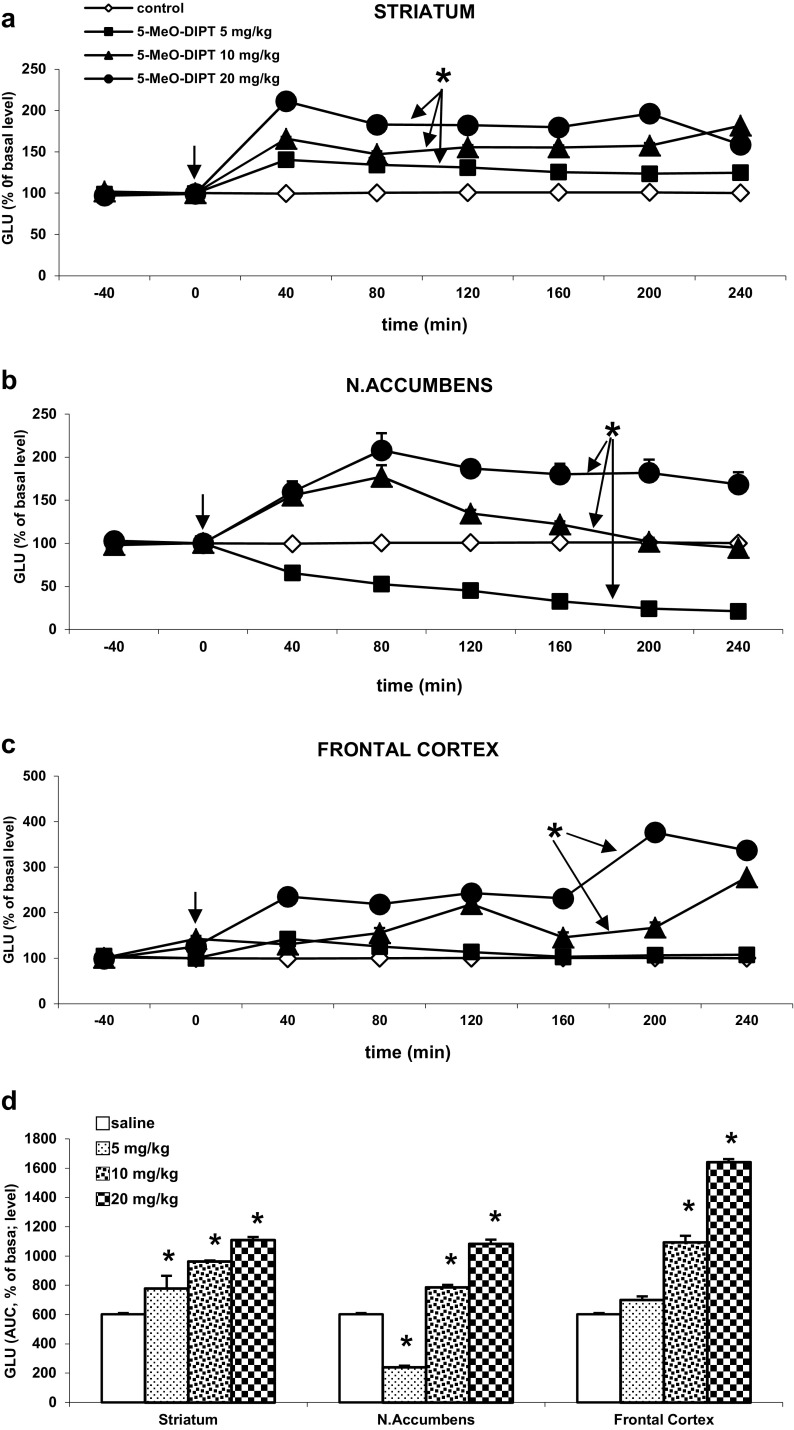
The effect of 5-MeO-DIPT (5, 10, and 20 mg/kg) on glutamate (GLU) release in the rat striatum (**a**), nucleus accumbens (**b**), and frontal cortex (**c**). Time-course effect is shown. Data are presented as the mean ± SEM (*n* = 4–7). Drug administration is indicated with an *arrow*. The total effect of 5-MeO-DIPT on GLU release (**d**) is expressed as an area under the curve (AUC) of the percent of basal level. *P < 0.01 in comparison to control group (repeated measures ANOVA and Tukey’s post hoc test)

5-MeO-DIPT significantly increased glutamate release in the striatum at all studied doses (*P* < 0.01, Fig. 4a). In the nucleus accumbens, the doses of 10–20 mg/kg 5-MeO-DIPT significantly enhanced extracellular glutamate level (*P* < 0.01); however, the lowest dose of 5 mg/kg had the opposite decreasing effect (*P* < 0.01, Fig. 4b). In the frontal cortex, only the doses of 10–20 mg/kg of 5-MeO-DIPT increased glutamate release (*P* < 0.01), while the dose of 5 mg/kg had no effect on extracellular glutamate level (Fig. 4c).

Repeated measures ANOVA showed a significant effect of treatment in the striatum F(3,15) = 486, *P* < 0, in the nucleus accumbens F(3,17) = 1279, *P* < 0, and in the frontal cortex F(3,15) = 259, *P* < 0; significant effect of time F(5,75) = 11, *P* < 0 in the striatum, F(5,85) = 33, *P* < 0 in the nucleus accumbens, and F(5,75) = 36, *P* < 0 in the frontal cortex. Interaction between both factors was also significant in the striatum F(15,75) = 14, *P* < 0, in the nucleus accumbens F(15,85) = 12, *P* < 0, and in the frontal cortex F(15,75) = 29, *P* < 0.

The total time-course effect of glutamate release in all studied brain regions defined as an area under the curve (AUC) is presented in Fig. 4d. The total effect of 5-MeO-DIPT shows a dose-dependent increase of glutamate release in the rat striatum. A similar effect was induced by 10–20 mg/kg 5-MeO-DIPT in the nucleus accumbens and frontal cortex. The lowest dose of 5 mg/kg 5-MeO-DIPT decreased or did not affect glutamate release in the nucleus accumbens and the frontal cortex, respectively (Fig. 4d).

### The Effect of 5-MeO-DIPT on the Contents of DA, 5-HT, and Their Metabolites in the Rat Striatum, Nucleus Accumbens, and Frontal Cortex

5-MeO-DIPT at all doses decreased DA, DOPAC, and HVA content in the rat striatum and at doses of 10–20 mg/kg in the nucleus accumbens and the frontal cortex (Table 1). The 5-HT tissue content was increased in the striatum and nucleus accumbens and remained unchanged in the frontal cortex. 5-HIAA tissue level was not affected by a dose of 5 mg/kg 5-MeO-DIPT, but was decreased by doses of 10–20 mg/kg 5-MeO-DIPT in all studied brain regions (Table 1).

**Table 1:** Tissue contents of DA, DOPAC, HVA, 5-HT, and 5-HIAA in the rat striatum, nucleus accumbens, and frontal cortex measured 4 h after administration of 5-MeO-DIPT

Treatement (mg/kg)	DA	DOPAC	HVA	5-HT	5-HIAA
Striatum pg/mg wt ± SEM (*n*)					
Control	12,876 ± 2018 (14)	1643 ± 239 (14)	1212 ± 276 (14)	456 ± 27 (14)	502 ± 24 (14)
5-MeO-DIPT 5	10,598 ± 915 (6)^a^	1217 ± 33 (6)^b^	732 ± 59 (6)^b^	745 ± 180 (6)^a^	510 ± 97 (6)
5-MeO-DIPT 10	7772 ± 723 (7)^b^	1120 ± 64 (7)^b^	683 ± 105 (7)^b^	576 ± 42 (7)^a^	406 ± 34 (7)^a^
5-MeO-DIPT 20	8295 ± 328 (10)^b^	1022 ± 55 (10)^b^	663 ± 46 (10)^b^	543 ± 30 (10)^a^	309 ± 45 (10)^a^
Nucleus accumbens pg/mg wt ± SEM (*n*)					
Control	13,787 ± 1663 (14)	2422 ± 262 (14)	1301 ± 144 (14)	794 ± 41 (14)	933 ± 130 (14)
5-MeO-DIPT 5	11,516 ± 1063 (6)	2241 ± 223 (6)	1140 ± 112 (6)	1155 ± 170 (6)^a^	830 ± 112 (6)
5-MeO-DIPT 10	7735 ± 591 (8)^b^	1770 ± 72 (8)^b^	708 ± 75 (8)^b^	1024 ± 111 (8)^a^	598 ± 85 (8)^a^
5-MeO-DIPT 20	7578 ± 589 (10)^b^	1606 ± 107 (10)^b^	643 ± 83 (10)^b^	1120 ± 118 (10)^a^	456 ± 43 (10)^a^
Frontal cortex pg/mg wt ± SEM (*n*)					
Control	567 ± 57 (14)	126 ± 14 (14)	130 ± 12 (14)	521 ± 50 (14)	240 ± 19 (14)
5-MeO-DIPT 5	535 ± 69 (6)	120 ± 6 (6)	100 ± 15 (6)	578 ± 96 (6)	230 ± 34 (6)
5-MeO-DIPT 10	390 ± 46 (8)^b^	102 ± 6 (8)^a^	85 ± 8 (8)^b^	524 ± 39 (8)	159 ± 12 (8)^a^
5-MeO-DIPT 20	344 ± 63 (10)^b^	101 ± 9 (10)^a^	80 ± 15 (10)^b^	573 ± 41 (10)	137 ± 12 (10)^a^

^a^ *P* < 0.05

^b^ *P* < 0.01 versus control group (one-way ANVA and Tukey’s post hoc test)

### The Effect of 5-MeO-DIPT on Oxidative Damage of Cortical DNA in Nuclei From the Rat Cortex

**Fig. 5:**
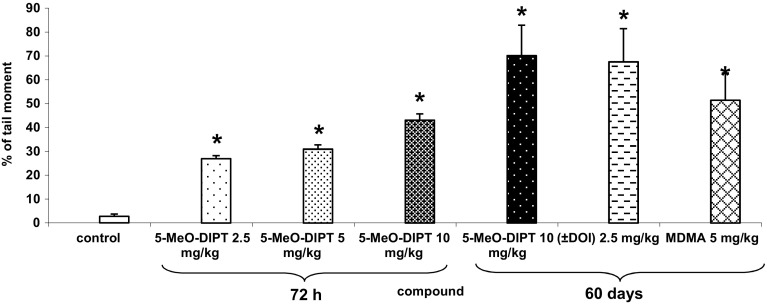
The effect of 5-MeO-DIPT (2.5, 5, and 10 mg/kg), MDMA (5 mg/kg) and (±)DOI (2.5 mg/kg) on the oxidative damage of cortical DNA in nuclei from the rat cortex. Data represent an olive tail moment. Olive Tail moment is defined as the product of the tail length and the fraction of total DNA in the tail. Tail moment incorporates a measure of both the smallest detectable size of migrating DNA (reflected in the comet tail length) and the number of damaged pieces (represented by the intensity of DNA in the tail). Loss of DNA integrity persisted from 72 h to 60 days after drugs administration. *P < 0.01 in comparison to control group (one-way ANOVA and post hoc Tukey’s test)

5-MeO-DIPT at single doses of 2.5, 5, and 10 mg/kg produced DNA damage shown as a percent of tail moment in the rat cortex 72 h after drug administration. The damage was greater 60 days after administration of 5-MeO-DIPT at the dose of 10 mg/kg. A similar effect was induced by (±)DOI at the dose of 2.5 mg/kg and was slightly weaker at 5 mg/kg of MDMA (Fig. 5).

## Discussion

The present study demonstrated a remarkable in vivo effect of 5-MeO-DIPT on brain neurotransmission by showing that 5-MeO-DIPT increased extracellular levels of DA, 5-HT, and glutamate in the rat striatum, nucleus accumbens, and frontal cortex. We also observed changes produced by 5-MeO-DIPT in tissue contents of DA and 5-HT as well as their metabolites DOPAC, HVA, and 5-HIAA in various regions of the rat brain. Furthermore, our data revealed a dose-dependent and progressive oxidative damage of cortical DNA by 5-MeO-DIPT. In addition, 5-MeO-DIPT evoked head twitches and potentiated forepaw treading induced by 8-OH-DPAT, which suggests activation of 5-HT2A and 5-HT1A receptors, respectively.

SERT inhibition by 5-MeO-DIPT (Blough et al. 2014) enhances 5-HT level which then affects all subtypes of serotonin receptors in the brain. In addition, 5-MeO-DIPT having by itself affinity for 5-HT1A, 5-HT2A, and 5-HT2C serotonin receptors (Fantegrossi et al. 2006) may potentiate the effects of endogenous serotonin. This interaction can lead to complex behavioral and neurochemical responses. In our study, 5-MeO-DIPT at the dose of 10 mg/kg elicited head twitches commonly used as a model of a hallucinogenic effect mediated through serotonin 5-HT2A receptors (Halberstadt 2015). The response to 5-MeO-DIPT (10 mg/kg) was similar in potency to the effect of selective 5-HT2A receptor agonist (±)DOI (2.5 mg/kg). In another animal model used in our work, 5-MeO-DIPT (5–10 mg/kg) strongly potentiated forepaw treading induced by 8-OH-DPAT, which is thought to be mediated via activation of postsynaptic 5-HT1A receptors (Sanchez et al. 1996; Sloviter et al. 1978). These data suggest that 5-MeO-DIPT enhances serotonin transmission in the brain and activates 5-HT1A and 5-HT2A receptors.

Blockade of intraneuronal serotonin transport by 5-MeO-DIPT led to a dose-dependent increase in extracellular 5-HT level in the rat striatum, nucleus accumbens, and frontal cortex as found in our study. Previous in vitro data of Sogawa et al. (2007) showed that micromolar concentrations of 5-MeO-DIPT inhibited [^3^H]5-HT uptake in COS cells transfected with SERT cDNA as well as in rat brain synaptosomes. The range of doses (5–20 mg/kg) used in our study seems to be effective in blocking SERT as submicromolar concentrations of 5-MeO-DIPT and its metabolites were found in rat urine samples after oral administration at the dose 5 mg/kg (Kanamori et al. 2006).

Enhancement of DA content in the mesocorticolimbic dopaminergic neurons is responsible for ability of several psychostimulant drugs to cause drug dependence and addiction. However, hallucinogens are not considered as reinforcing drugs (O’Brien^2001^). In contrast to LSD, 5-MeO-DIPT, like other tryptamines (e.g., 5-MeO-DMT), does not display affinity for dopamine receptors and has a low activity in blocking dopamine transporter DAT (Halberstadt and Geyer 2011; Sogawa et al. 2007). Nevertheless, we show evidence that 5-MeO-DIPT at doses of 10–20 mg/kg is able to increase DA release in the striatum, nucleus accumbens, and frontal cortex; however, at a dose of 5 mg/kg, it was less effective. The possible mechanism responsible for this activity of 5-MeO-DIPT in increasing DA release may be related to the 5-MeO-DIPT-induced stimulation of presynaptic 5-HT2A receptors located on DA neuronal terminals. The data supporting our results were reported by Pehek et al. (2001) who showed that a stimulation of DA release by potassium in the rat prefrontal cortex was mediated by 5-HT2A receptors. Other researchers demonstrated that the effect of 5-HT2A agonist (±)DOI on DA release in the rat nucleus accumbens and the rat striatum was antagonized by 5-HT2A antagonists ketanserin (Yan 2000) or SR 46349B (Lucas and Spampinato 2000). Alternatively, enhancement of DA release by 5-MeO-DIPT may be mediated through the activation of somatodendritic 5-HT2A receptors in the VTA. Those receptors might directly affect local dendritic release of DA and subsequently increase extracellular DA level in mesolimbic or mesocortical DA terminals as suggested by Celada et al. (2001) and Vazquez-Borsetti et al. (2009). Moreover, high affinity of tryptamine hallucinogens for 5-HT1A receptors reported by deMontigny and Aghajanian (1977), and Titeler et al. (1988) suggests that these receptors may play a role in controlling activity of DA neurons. 5-HT1A receptors localized on GABA-ergic interneurons in limbic and cortical brain regions (Hamon et al. 1990; Pazos and Palacios 1985) may disinhibit descending glutamatergic pathways which can subsequently stimulate DA cells. The data presented by Tanda et al. (1994), Sakaue et al. (2000), and Wędzony et al. (1996) support our conclusion, as they demonstrated that selective 5-HT1A receptor agonists, *R*(+)-8-OH-DPAT or ipsapirone, increased DA release in the frontal cortex.

We found that 5-MeO-DIPT increased extracellular glutamate level in the striatum at all doses and only at higher doses in the nucleus accumbens and frontal cortex. The enhancement of glutamate release by 5-MeO-DIPT may depend on activation of several subtypes of serotonin receptors, and therefore may vary between brain regions. As noted by other researchers, 5-MeO-DIPT acting at postsynaptic 5-HT2A receptors on pyramidal cells enhances glutamate release (Beique et al. 2007). However, 5-HT2A receptors are co-localized on cortical pyramidal cells with serotonin 5-HT1A receptors (Martin-Ruiz et al. 2001), where the two receptor types have opposing effects (Araneda and Andrade 1991). In our study, the decrease in glutamate release caused by the lowest dose of 5-MeO-DIPT in the nucleus accumbens or lack of effect in the frontal cortex suggests that 5-MeO-DIPT at small concentrations preferentially activates 5-HT1A receptors, causes inhibition of pyramidal cells, and subsequently decreases glutamate release. At higher doses, the effect exerted by 5-HT1A receptors is opposed by 5-HT2A receptors, which results in the stimulation of glutamate release. In fact, in vitro affinity of 5-MeO-DIPT at 5-HT1A receptors was found in nM, while at 5-HT2A receptors in μM range of concentrations (Fantegrossi et al. 2006). Therefore, the effect mediated via 5-HT1A receptor may be counteracted by 5-HT2A receptor activated by higher concentration of 5-MeO-DIPT.

The finding that hallucinogens act as agonists of 5-HT2C receptor suggests that these compounds exert some effects via the 5-HT2C receptor subtype. However, there is now a consensus that ability of (±)DOI to induce head-twitch response is not blocked by 5-HT2A/C antagonists (Fantegrossi et al. 2010; Schreiber et al. 1995; Wettstein et al. 1999). It also appears that activity at the 5-HT2C receptor attenuates many of the behavioral effects of hallucinogens. For instance, the ability of (±)DOI to reduce prepulse inhibition in rats was reversed by the 5-HT2C selective agonist WAY-163,909 (Marquis et al. 2007). Furthermore, Halberstadt et al. (2009) demonstrated that 5-HT2A and 5-HT2C receptors exerted opposing effects on locomotor activity in mice. Similar findings have been reported for head-twitch response in mice (Fantegrossi et al. 2010) or in rats (Vickers et al. 2001). Therefore, some effects observed in our study, such as a decrease in DA or glutamate release by a low dose of 5-MeO-DIPT in the striatum or in the nucleus accumbens, respectively, may result from a modulating role of 5-HT2C receptor. However, exact mechanism of the interaction between serotonin receptor subtypes in their effect on brain neurotransmission needs further studies.

In terms of the 5-MeO-DIPT effect on 5-HT tissue content, our data indicate that an increase or lack of changes in serotonin level and a decrease in 5-HIAA level are consistent with the hypothesis that 5-MeO-DIPT, through blocking intraneuronal transport of 5-HT inhibits serotonin metabolism in all studied brain regions. The observed decrease in DA, DOPAC, and HVA tissue levels by all doses of 5-MeO-DIPT in the rat striatum and two higher does in the nucleus accumbens and the frontal cortex appears to be related to a feedback inhibition of DA synthesis as a response to stimulation of dopamine receptors by an increased synaptic pool of DA. On the other hand, a deficit in tissue content of DA and its metabolites may be associated with neurotoxic effect exerted by 5-MeO-DIPT on presynaptic DA terminals.

The possible neurotoxic effects of 5-MeO-DIPT seem to be supported by our findings obtained with the use of the comet assay. It was demonstrated that 5-MeO-DIPT given at a single dose produced DNA single and double-strand breaks in the rat cortex. The magnitude of tail moment reflecting the extent of DNA damage was time- and dose-dependent when measured 72 h and 60 days after administration. A similar effect on DNA damage was observed after treatment of rats with the 5-HT2A agonist (±)DOI and MDMA. The oxidative damage of DNA was reported in brains of animals treated chronically with high doses of MDMA and methamphetamine (Frenzilli et al. 2007; Johnson et al. 2015). The mechanism of DNA oxidation by amphetamine derivatives is related to an oxidative stress and the formation of highly reactive free radicals. Excessive release of DA and glutamate by MDMA or methamphetamine leads to formation of reactive oxygen and nitrogen species as well as reactive quinones, which can damage DNA (Halliwell and Whiteman 2004). Our study is the first to show genotoxic effect of a tryptamine hallucinogen. An increase in DA and glutamate release by 5-MeO-DIPT reported in the present study suggests that DA and glutamate play a role in the induction of oxidative stress. However, other factors such as protective mechanisms and levels of antioxidants which control free radical generation, may also be affected by 5-MeO-DIPT. Therefore, further studies are needed to elucidate the possible mechanism of 5-MeO-DIPT genotoxicity. It has to be emphasized that oxidative stress which has been implicated in neurodegenerative diseases, such as Parkinson’s and Alzheimer’s diseases (Fahn and Sulzer 2004; Halliwell 2006) and which causes oxidative DNA damage seems to be linked with memory loss and cognitive dysfunction in rats (Liu et al. 2002). Alteration in the ability of rats to perform certain cognitive tasks in Cincinnati water maze (Williams et al. 2007) or impairments in working memory (Compton et al. 2011) by 5-MeO-DIPT may be due to persistent and progressive modification of the cortical cell function. All these observations suggest that tryptamine hallucinogens need further extensive studies as they are among the most popular groups of illicit drugs.

In summary, the results of our study demonstrate that exposure of rats to the tryptamine hallucinogen 5-MeO-DIPT produces changes in extracellular serotonin, dopamine, and glutamate levels in cortical and subcortical rat brain regions. Our findings also support the conclusion that hallucinations after administration of tryptamine analogues may be mediated by changes in glutamatergic neurotransmission. The progressive oxidative damage of DNA produced by a single dose of 5-MeO-DIPT indicates development of oxidative stress and suggests marked neurotoxicity of this drug.
